# Universal Stokes’s nanomechanical viscometer

**DOI:** 10.1038/s41598-021-93729-0

**Published:** 2021-07-13

**Authors:** Komal Chaudhary, Pooja Munjal, Kamal P. Singh

**Affiliations:** Department of Physical Sciences, Indian Institute of Science Education and Research, Mohali, Knowledge City, Sector 81, Manauli, 140306 India

**Keywords:** Energy science and technology, Engineering, Nanoscience and technology, Optics and photonics, Physics

## Abstract

Although, many conventional approaches have been used to measure viscosity of fluids, most methods do not allow non-contact, rapid measurements on small sample volume and have universal applicability to all fluids. Here, we demonstrate a simple yet universal viscometer, as proposed by Stokes more than a century ago, exploiting damping of capillary waves generated electrically and probed optically with sub-nanoscale precision. Using a low electric field local actuation of fluids we generate quasi-monochromatic propagating capillary waves and employ a pair of single-lens based compact interferometers to measure attenuation of capillary waves in real-time. Our setup allows rapid measurement of viscosity of a wide variety of polar, non-polar, transparent, opaque, thin or thick fluids having viscosity values varying over four orders of magnitude from $10^{0}{-}10^{4}~\text{mPa} \, \text{s}$. Furthermore, we discuss two additional damping mechanisms for nanomechanical capillary waves caused by bottom friction and top nano-layer appearing in micro-litre droplets. Such self-stabilized droplets when coupled with precision interferometers form interesting microscopic platform for picomechanical optofluidics for fundamental, industrial and medical applications.

## Introduction

More than a century ago Stokes suggested a conceptually elegant and universal idea to determine viscosity of a fluid by exploiting damping of nanomechanical capillary waves^1^. However, a few attempts were made to realize Stokes universal idea owing to challenges in controlled generation and precise detection of nano-mechanical capillary waves on arbitrary fluids. Using a fibre-optics probe viscosity of bulk water was measured by generating standing capillary waves in a feet-long container^2^. Besides, the fiber-optics interferometer had limited working distance, lacked nanoscale precision, and importantly, could not measure viscosity of colored fluids as well as of highly viscous honey-like samples.

Besides requiring large quantity of fluids, conventional contact-based techniques are slow. For example, by letting fluids flow through thin capillaries or by tracking objects falling through a stationary fluid the measurement time depends on the viscous drag and may become several hours for highly viscous fluids^3^.

Many non-contact optical viscometers based on optical tweezers^4–8^, micro-fluidics^9–11^, and laser induced surface deformation^12^ have also been devised. Methods based on optical tweezers determine the viscosity at micrometer scale by analyzing the motion of optically confined micro-particles in the liquid^4–8^. Technique of laser induced surface deformation measures the viscosity using time delay in liquid response following impulse laser irradiation^12^. Although, all-optical excitation of fluid interface using radiation-pressure and optical detection of surface dynamics is attractive, these approaches are limited to semi-transparent samples, because otherwise the laser absorption essentially induces thermal effects which compete with viscous damping. Several techniques have been used to study surface waves on liquids such as photon correlation spectroscopy (PCS)^13^ of thermal fluctuations, light scattering^14,15^, diffraction from dynamic grating on fluid surface^16–18^, imaging^19–21^, profilometry^22,23^ and laser interferometry^24,25^. In photon-correlation spectroscopy technique, although thermal fluctuation can excite surface waves on most fluids, measuring attenuation using line broadening of the scattered laser light requires integration time of about 20 s and therefore, cannot measure nanomechanical deformation in real-time.

Here, we demonstrate a universal Stoke’s viscometer by electrically exciting quasi-monochromatic capillary wave of desired frequency and optically probing its propagation and attenuation with sub-nanometer precision simultaneously at two distinct points using a pair of compact laser interferometers. We show easy and rapid measurements of viscosity of a wide variety of polar, non-polar, transparent to opaque, thin or thick fluids of viscosity spanning four orders of magnitude from $10^{0}{-}10^{4}~\text{mPa}\,\text{s}$, in agreement with their known values. Furthermore, for micro-liter sessile fluid droplets, we demonstrate enhanced damping of surface waves due to bottom friction and adsorbed top layer, compared to the bulk fluid, suggesting that such self-stable droplets when coupled with precision interferometers makes an attractive microscopic platform for picomechanical optofluidics.

## Working principle

Stokes described attenuation of a propagating surface capillary wave of wavelength $\lambda$ in terms of the mechanical properties of fluid like density and viscosity. The viscosity is related to the rate of energy dissipation in a propagating wave as^1, 2^

1 $$\begin{aligned} dE/dt=-(4 k^{2} \eta / \rho ) E \end{aligned}$$

where E is wave energy per unit area, $k=2 \pi /\lambda$ is wave number, $\eta$ is dynamic viscosity and $\rho$ is the liquid density. As the wave energy is directly proportional to the square of the wave amplitude *a*, Eq. (1) can be rewritten as

2 $$\begin{aligned} da/a=-(2 k^{2} \eta / \rho ) \Delta x/v_g \end{aligned}$$

where $v_g$ is the group velocity of wave and $\Delta x =v_g dt$ is the distance travelled by the wave in time *dt*. Equation (2) can be further transformed as

3 $$\begin{aligned} a=a_{0}e^{-\alpha x} \end{aligned}$$

where $a_{0}$ is the initial wave amplitude at $x=0$ and $\alpha =(2k^{2}\eta )/\rho v_g$ is the attenuation coefficient defined as the inverse of distance over which the initial amplitude decays to 1/*e*. Thus the expression of viscosity can be written as

4 $$\begin{aligned} \eta =\dfrac{\alpha \rho v_g}{2k^{2}} \end{aligned}$$

By measuring $v_g$ and $\alpha$ using the simultaneous two-point detection in our setup, (*k* is experimental parameter and $\rho$ is known) the viscosity $\eta$ can be precisely determined from the Eq. (4). The precision in $\eta$ is governed by the uncertainty in the measurements of probe position and wave amplitude, which is estimated to be $0.01~\text{mPa} \, \text{s}$ using error propagation in our measurements.

## Results

### Methodology

**Figure 1:**
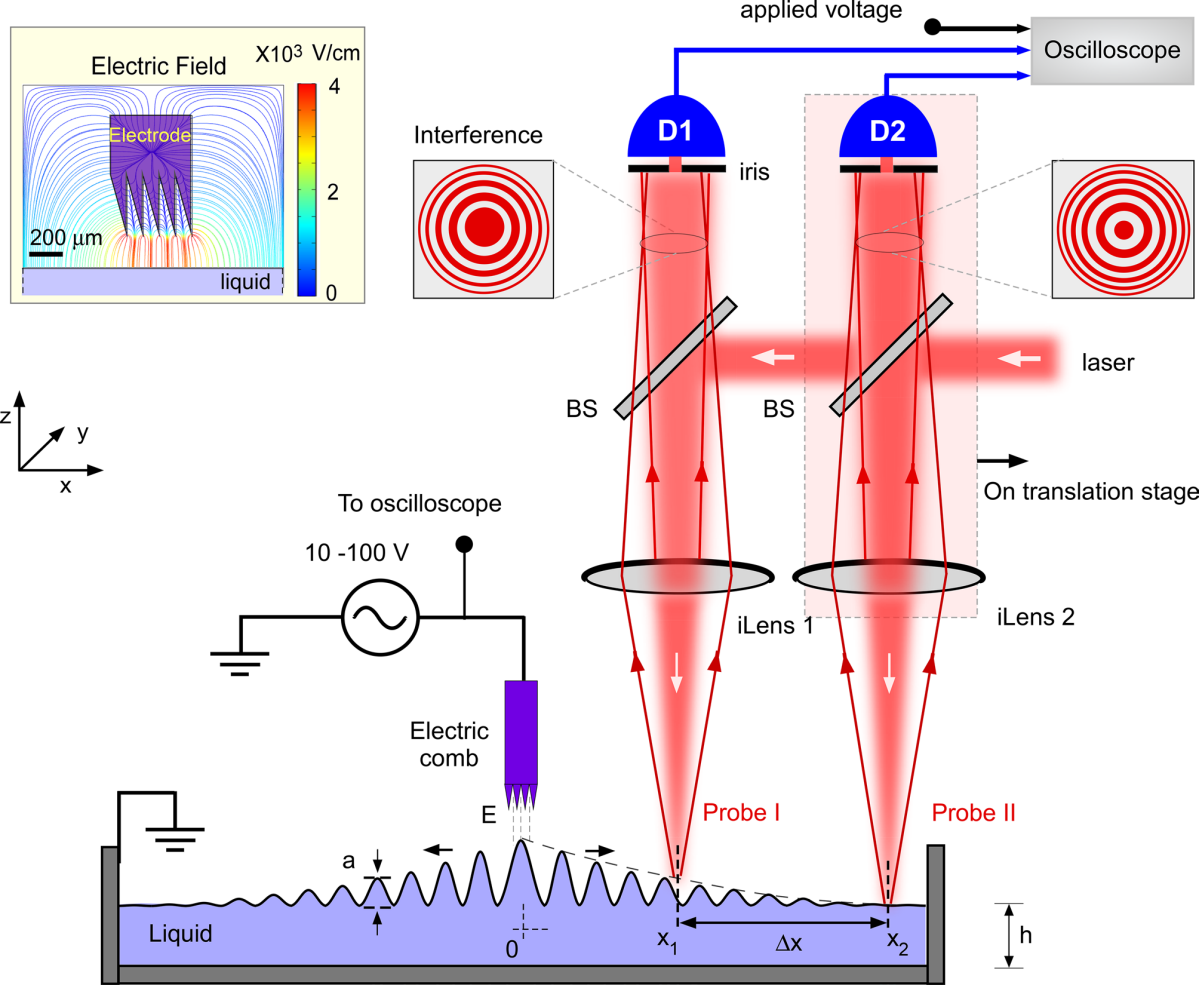
Schematic of experimental setup for viscosity measurement from capillary waves. Capillary waves were generated electrically with multiple sharp edges and their dynamics was resolved by two simple and compact interferometric probes. High-contrast circular interference fringes were obtained by combining the reflections from lenses and liquid surface. Photo-diodes (D1 and D2) record central fringe intensity in oscilloscope. iLens1,2: single-lens interferometers, BS: Beam splitter. Inset presents the simulated electric field of the electrode at 50 V. The schematic drawing is created in Canvas X, Version: X Built 925. https://www.canvasgfx.com/.

Our experimental setup is shown in Fig. 1. Liquid sample ($\sim 30$ ml) was filled in a clean glass container of rectangular base to excite capillary wave mostly along the x-axis. The height of the liquid ($h \sim 1$ cm) was chosen to satisfy the deep-fluid limit, where $h > 0.5\lambda$, for a typical wavelength of the capillary wave in the range of ($\lambda = 0.2{-}1$ cm). This parameter regime also avoids additional damping due to frictional contribution from the bottom surface of the container. The sample volume can be further reduced to a few ml by reducing the base size of the container and by miniaturizing iLens and the electrode.

Capillary surface waves were generated electrically using a comb-like electrode consisting of multiple knife-edges with sharpness of each edge about 250 nm (see the supplementary Fig. S1 for the electron microscope image). Our design of the electrode enhanced the electric field on the fluid-surface to adequately actuate the fluid with low-voltage driving $0{-}50~\text{V}$. The electrode was placed a few tenths of millimeter ($\sim 100{-}200~\upmu \text{m}$) above the liquid surface and total width of the electrode $\delta w =500~\upmu \text{m})$ was much smaller than the excited wavelength. The spacing between the electrode and liquid is experimentally optimized to be as close as possible to the fluid surface for detectable actuation without causing any short-circuiting due to oscillating fluid surface. To excite quasi-monochromatic capillary waves, the electrode was fed with a sinusoidal voltage at desired fixed frequency in the range $(10{-}160~\text{Hz})$. Application of a voltage difference between the electrode and fluid produced a strong electric field ($\sim 1000~\text{V}/\text{cm}$) below the electrode and normal to the fluid surface. Comsol simulation of the electric potential and electric field lines for the experimental geometry is shown in inset of Fig. 1 and further detailed in supplementary Fig. S2. Application of the sinusoidal local excitation generates the travelling surface waves at the exact same frequency which then propagate away from the source. As the transverse length of the blade (in y direction) is much larger than its sharpness $\delta w$, the wave propagation is mostly along the length of the container (in $\pm x$-direction). The amplitude of the surface waves is experimentally adjusted in the range of a few micrometers to $sub-100~\text{nm}$ by controlling the applied voltage or by tuning the electrode-fluid distance. The amplitudes of the capillary waves are much smaller than the excited wavelengths $(a<0.001\lambda )$, in agreement with the limit of validity of theoretical solutions, to avoid any non-linear effects on wave dispersion and attenuation which may arise for large amplitude excitation^26,27^. It is worth mentioning that our electrical actuation works well for all the tested liquids, including weakly (non-)polar fluids since the fluid surfaces can be polarized by the oscillating electric field leading to detectable capillary surface waves.

To measure nanomechanical propagation and attenuation of the capillary surface waves, we measured the deformation of fluid’s surface at two distinct points with a pair of single interference lens (iLens) based universal interferometers. The iLens interferometer was previously shown to offer about 20 picometer precision on arbitrary solid surfaces^28^. However, its applicability to probe fluid surface was not previously demonstrated. A He-Ne laser (632 nm, 1 mW on the fluid surface) was loosely focused $(2\omega _0=100~\upmu \text{m})$ on the liquid surface by the iLens and the two partial reflections, one from the upper surface of the iLens (reference beam) and second from the air-fluid surface were made to interfere on screen. In addition to being a compact and self-calibrating probe, the iLens readily produced Newton’s ring like high-contrast interference fringes from all the tested fluids, including smooth transparent fluid (water) to black diffusive liquid (black ink). A pair of iLens probes were separated by a horizontal distance $\Delta x$ and the iLens allowed a large working distance of about 5 cm from the sample surface. One of the interferometric probe (Probe-II, see Fig. 1) was attached to a linear translation stage having a step size of $0.05~\upmu \text{m}$ and 5 cm horizontal travel to control the distance between the two probes.

To measure the attenuation of propagating surface wave in real-time, we measured wave amplitudes at two points $x_1$ and $x_2$ simultaneously using iLens1 and iLens2. We measured the central fringe intensities $I_{D1}$ and $I_{D2}$ independently using a pair of pinholes and fast photodiodes $D_1$ and $D_2$, respectively, along with the driving electrode voltage. The size of pin hole was typically below 1 mm and was kept much smaller than the width of the central fringe on the detector plane. An oscillating liquid surface varied the optical lengths between iLens1,2 and the fluid surfaces at $x_{1,2}$ and produced corresponding variation in the PD signals which was used to directly measure the wave amplitudes at the two probe points. The central fringe intensity provides a self-calibrated measurement of surface displacement. Briefly, as the transmitted ray travels twice the air-gap between the ilens and liquid surface, liquid displacement of $\lambda _{l}/4n=158~\text{nm}$, where $\lambda _{l}$ is laser wavelength and *n* is refractive index of air-gap, would lead to the evolution of half fringe. The ambient temperature was measured to be $20\pm 0.5\;^{\circ} \text{C}$ with $RH\sim 55\pm 5\%$ during the experiment.

### Viscometry from capillary wave damping

**Figure 2:**
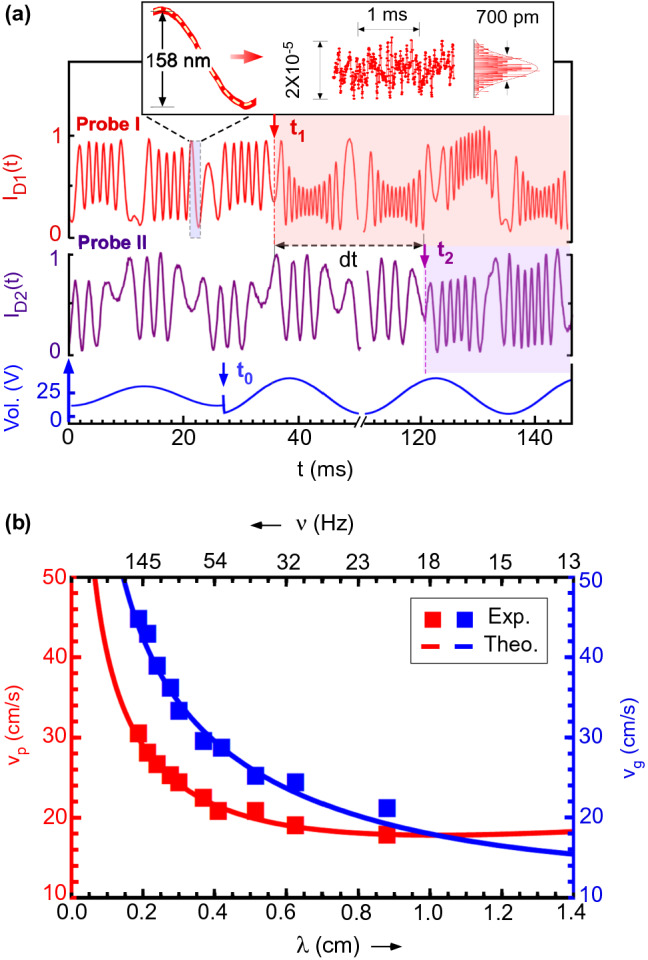
Propagation and dispersion of capillary waves on milk. (**a**) Central fringe intensities $I_{D1}$ and $I_{D2}$ of interferometric probes, separated by a distance ($\Delta x=2$ cm along with the periodic driving voltage at 40 Hz. Inset shows the precision of our interferometer in terms of the residual noise $\sim 700~\text{pm}$. The residual noise is estimated after subtracting background oscillations over half-cycle (dotted yellow line). (**b**) Capillary wave velocities at different excitation frequencies for milk. Blue and red squares are the measured values and solid lines are the theoretical fits. Error bars are within the size of data points.

Figure 2a shows our measurements on a sample of milk actuated at 40 Hz. The signals for the central fringe intensities of the two interferometric probes, separated by a distance $\Delta x = 2~\text{cm}$, is shown along with the sinusoidal applied voltage (see Fig. S3 for detailed data). We observed that in response to sinusoidal voltage, capillary waves are produced on the milk’s surface at the same frequency. Oscillating fluid surface periodically varies the path lengths between the iLens and the liquid surface causing the interference fringes to evolve as shown in Fig. 2a. During positive half cycle, an increase in the applied voltage pulls the fluid surface upward causing interference fringes to evolve outwards which collapse back with the fall in voltage. The rate of evolution of fringe follows the sinusoidal waveform corresponding to the propagating capillary waves. Fringes evolve uniformly in the linear regime and become slow near trough and crest of wave. As the wave amplitude decays exponentially with propagation distance, the number of fringes evolved in the second probe is reduced when compared to the first probe. We measured the amplitude of surface waves by counting the number of fringes moved in one direction and equating the half fringe collapse to 158 nm. Our detection technique offers sub-nanometer resolution to detect nano-mechanical waves with pm precision. Inset shows the experimental noise floor of $\sim 700~\text{pm}$ which is calculated after subtracting the sinusoidal baseline^28–30^.

Attenuation coefficient $\alpha$ was readily calculated by measured amplitudes at two distinct points using $\alpha =ln(a_{1}/a_{2})/(x_{2}-x_{1})$ where $a_{1}$ and $a_{2}$ are wave amplitudes at $x_{1}$ and $x_{2}$ probe positions, respectively. The simultaneous two-points measurement scheme was further used to measure the (group) velocity by measuring the time delay between the signals detected at the two probe positions when a step-like sudden increment is applied to the amplitude of the oscillating voltage, which served as a reference event. The wave-event with enhanced amplitude arrives first at the nearest Probe-I at a time $t_1$ and reaches the Probe-II at a later time $t_2$ since it is farther away by a distance $x_2-x_1$. The propagation delay *dt* was measured with fast photodiodes with rise time of 1 ns. The second probe rested on a linear translation stage (dotted enclosure in Fig. 1) and its horizontal displacement was measured with $0.1~\upmu \text{m}$ precision. By measuring the separation $\Delta x$ between two probes and the delay time *dt*, the group velocity was calculated. Our measured velocity values nicely match with the theoretically expected values for group velocities $v_g=d\omega /dk$ (Fig.2b) obtained from dispersion relation of surface waves in capillary regime^31^, $\omega ^2=gk+[\sigma /\rho -(8\eta ^3\omega /\rho ^3)^{1/2}+4k\eta ^2/\rho ^2]k^3$ where $\sigma$ is the surface tension of the fluid. Furthermore, for a chosen oscillation frequency, the wavelength of the capillary waves was measured by translating the probe and observing the relative phase difference between the applied voltage and the PD probe signal. The measured phase velocity $v_p$ of capillary waves for various excitation frequencies agreed well with the theoretical relation $v_p=\omega /k$ with $\omega$ and *k* being the angular frequency and wave-number of the surface waves respectively. Using the measured attenuation and velocity values, we calculated the dynamic viscosity of the milk ($2.00\pm 0.30~\text{mPa} \, \text{s}$). We also cross validated our result by measuring the same sample using a traditional capillary viscometer by letting the milk to flow through a thin capillary (1  mm diameter and 10  cm channel length) under gravity action and with the values reported in literature^32^. Our result matched with the expected value of the viscosity with an added advantages of being simple, fast and non-invasive.

**Figure 3:**
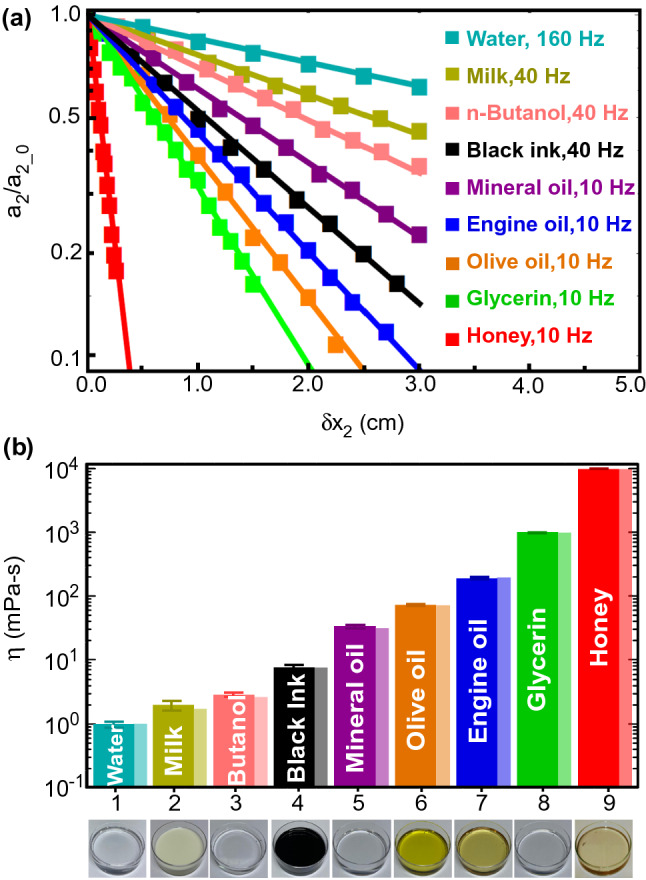
Damping of capillary waves and viscosity measurements. (**a**) Exponential decaying normalized amplitude of surface waves (on logarithm scale) with propagation distance $\delta x_2 = x_2-x_{2\_0}$ for various tested liquid samples. $x_{2\_0}$ is the initial position of probe II. Less viscous fluids were excited at higher frequencies as the attenuation is much small at lower frequencies. (**b**) Measured (darker color bars) and expected (lighter color bars) viscosity values for all tested samples. Images of tested liquids of different opacity are also shown.

We demonstrate wide applicability of our techniques by measuring the dynamic viscosity of various industrial and laboratory liquids including water, 1-butanol, black-ink, mineral oil, olive oil, engine oil, glycerine and honey with a wide range of viscosities spanning over four orders of magnitude from $10^{0}{-}10^{4}$ mPa s. The Stokes viscometer offered a unique advantage of rapid measurement time due to intrinsically fast damping of microscopic waves on most samples. Our interferometric probes produced high-contrast fringes from a variety of transparent and opaque liquids even from highly black diffusive surfaces. Images of tested liquids with different opacity are also shown in Fig. 3. Figure 3a shows exponential decay of normalized surface wave amplitude ($a_2/a_{2\_0}$) with probe position $x_2$ for various test liquids. $a_2$ and $a_{2\_0}$ are wave amplitudes at second probe $x_2$ with respect to its initial position at $x_{2\_0}$, respectively. The propagation distance $x_2$ was scanned with the moving probe iLens2. Different range of excitation frequencies was chosen for thick and thin liquids. Less viscous fluids were excited at higher frequencies as the attenuation is very less at lower frequencies. It is worth mentioning that the waves in thick viscous fluids damp rapidly with the propagation distance. We plotted the measured viscosity values (in darker color bars) for all tested liquid samples in Fig. 3b and our results have a good agreement with expected values (in lighter color bars). Furthermore, we cross-checked our results with viscosity obtained with traditional contact-based capillary viscometer. Table 1 shows viscosity values measured with our technique (measured) and standard capillary viscometer (expected) for all samples with literature references. Apart from the multiple repetition for accurate results, measurements with capillary viscometer are time consuming. High viscous fluids such as glycerin, honey may take several hours to flow through capillary viscometer. Besides, cleaning of the capillary is crucial to avoid contamination and is tedious to perform before new measurement. In contrast, with our technique even thick fluids like glycerin and honey, the microscale capillary waves can be readily excited electrically and precisely detected in real-time at large working distance with the probe-pair separated by a few mm. It is also worth mentioning that for the used moderate electric fields we did not observe any evidence of heating in the fluid or any electric breakdown of the samples during the experiments.

**Table 1:** Comparison of viscosity of various samples measured with present method and against capillary viscometer and the values reported in literature.

Liquid	Measured viscosity (mPa S)		
	Stokes’ viscometery	Capillary viscometer	Literature references
Water	$0.99\pm 0.01$	$1.00\pm 0.1$	$\sim 1.0$^2^
Milk	$2.02\pm 0.30$	$1.75\pm 0.3$	$\sim 1.8$^32^
n-Butanol	$2.83\pm 0.20$	$2.62\pm 0.2$	$\sim 2.8$^33^
Black fountain pen ink	$7.60\pm 1.00$	$7.00\pm 1.0$	$\sim 5.8$^34^
Mineral oil (n20/D)	$32.09\pm 2.00$	$30.00\pm 2.0$	$\sim 30$^35^
Olive oil	$71.65\pm 2.00$	$70.00\pm 2.0$	$\sim 72$^36^
Engine oil (10W-40)	$186.62\pm 5.00$	$188.00\pm 5.0$	$\sim 200$^37^
Glycerin	$947.54\pm 10.00$	$940.00\pm 10.0$	$\sim 1000$^38^
Honey	$9982.78\pm 50.00$	Can’t measure	$\sim 10{,}000$^39^

### Additional damping mechanisms: bottom friction and surface adsorption

Two additional mechanisms causing damping of a propagating capillary wave are worth discussing. One due to friction arising from the bottom surface and second arising from contamination layers, if any, present on the fluid surface. It is important to differentiate these different regimes of wave attenuation to further miniaturize the setup as well as to understand important precaution to make clean measurement.

When the sample volume is further reduced such that the liquid depth (h) becomes less than the wavelength of the capillary wave, i.e. for $h<0.5\lambda$, the surface wave induces a tangential flow near the bottom surface of the rigid substrate. Due to the bottom friction a vertical velocity gradient is produced within the boundary layer, keeping zero velocity at the bottom surface, which affects the wave propagation and attenuation. The propagation of the wave in the presence of boundary layer can be solved using the concept of displacement thickness and the proportional loss of wave energy per oscillation period due to the bottom friction is given by $dE/E=(4\pi )(\eta /2\omega \rho )^{1/2}(k/\sin h[2kh])$ where $\omega$ is angular frequency^1^. For small *h*, the bottom friction adds non-negligible correction to the damping of the surface waves given previously by Eq. (1)). One can verify that for large *h*, energy dissipation due to bottom friction vanishes and the remaining dissipation agrees with the internal dissipation considered previously (Eq. (1)). The bottom friction becomes dominant for μL sample volumes (or small droplets) where the liquid height is fairly small.

**Figure 4:**
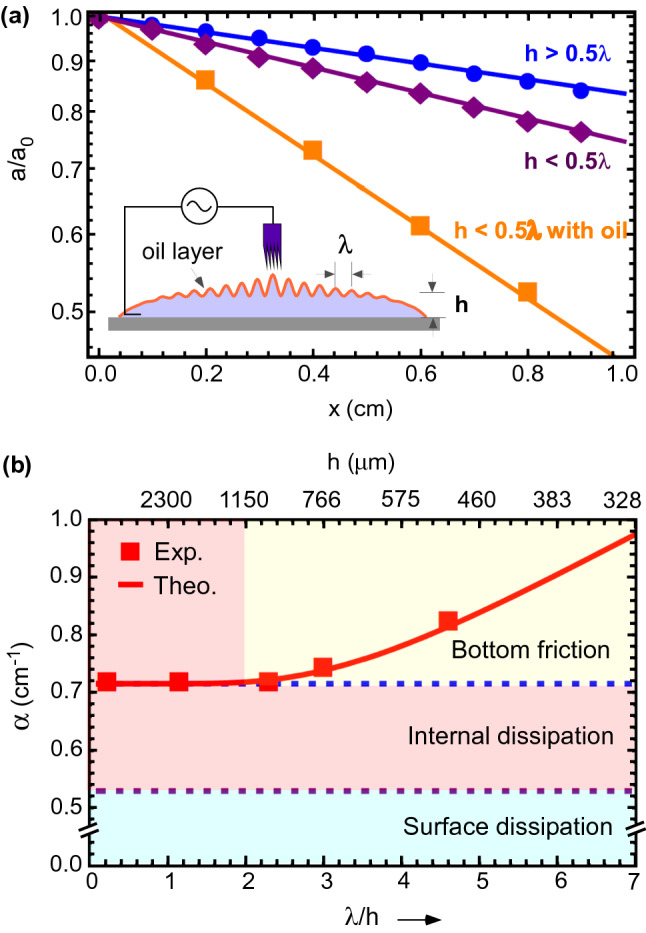
Manifestation of three processes of wave attenuation. (**a**) Exponential decay of wave amplitude in deep water $h>0.5\lambda$, shallow water $h<0.5\lambda$ and shallow water covered with thin layer of mustard oil at 160 Hz frequency. Inset illustrates the studied geometry. (**b**) Measured and theoretical values of wave attenuation coefficient, $\alpha$, is plotted with shaded region of different contributions of energy dissipation. Error bars are within the size of data points (red square).

These three mechanisms of damping of capillary waves are isolated for a sessile water drop in Fig. 4a. The drop was carefully placed on a clean glass surface such that it had a large footprint diameter of $\sim 2.5~\text{cm}$ (much larger than the capillary length $\sim 2.7$ mm). This ensures that the air-water interface remains almost flat near the center of the drop, except near the contact boundary. We excited surface waves of $\lambda =2.3~\text{mm}$ ($\nu = 160~\text{Hz}$) at the centre of different droplets with different water heights. For $h>0.5\lambda$, water behaves as deep water where the bottom friction can be neglected and measured dissipation is only because of internal viscous forces. On reducing the water depth to $(h<0.5\lambda )$, bottom friction comes into picture and an increase in wave damping is observed. Surface contamination can affect the surface dissipation due to departure in surface tension of pristine fluid. To show this effect, we covered water surface with a nanometric layer of mustard oil as shown in the inset of Fig. 4a (estimated thickness about 500 nm). We observed that for droplet covered with oil, the waves damping was significantly higher. Water depths and thickness of oil layer, both were calculated from known volume of liquid spread in a certain base area. Measured and theoretical values of wave attenuation coefficient, $\alpha$, is plotted in Fig. 4b with shaded region of different contributions of energy dissipation. Water has very low viscosity and adding a layer of oil on it remarkably increases the wave attenuation. This effect is known as the calming effect of oil on water. Our results are consistent with the attenuation data reported in literature for oil layer on the water^40^. These demonstrations suggest that one must carefully avoid surface contamination as well as choose appropriate water depth for clean measurement of viscosity.

## Conclusions

We demonstrate a universal, non-contact electro-optical setup to rapidly measure the viscosity from nanomechanical damping of surface capillary waves. Our technique resolves nanomechanical dynamics of surface waves with *pm* precision. Our technique is applicable for a wide variety of fluids including transparent, colloidal solutions, even highly absorbing black liquids having viscosity ranging from $10^{0}{-}10^{4}$ mPa s. Furthermore, we isolate additional damping caused by bottom friction for tiny droplets as well as issue of contamination of the sample in viscosity measurement. Enhanced damping significantly eliminated fluctuations in thin sessile liquid drops suggesting that such self stable drops, coupled with optical interferomters, form an attractive microscopic platform to study intriguing interface phenomenon with unprecedented picometer scale resolution^29,30^. Isolation of three distinct mechanisms of wave damping, especially differentiating bottom friction from internal viscous stresses opens a route to further miniaturize the setup to determine the viscosity with micro-liter fluid samples. It will be interesting to probe viscosity of complex biological fluids and gels, non-Newtonian effects and their evolution when such systems are subjected to external electric, thermal or pressure fields^41^.

## Methods

### Sample preparation

The experiments were performed in a clean rectangular glass container of dimension $10~\text{cm} \times 3~\text{cm}\times 1.5~\text{cm}$. Container was washed 4–5 times with water and bleaching agents, and kept for sometime to be completely dry every time before using to avoid any contamination issue. Sample liquids were directly poured in the container without any external contact. For a reference, viscosity values of tested liquids were also calculated using capillary viscometer by measuring the time that they take to flow through a thin capillary.

### Electrode fabrication

To excite the nanomechanical waves, electrode was designed with 5 sharp metallic blades of sharpness 250 nm and thickness of $100~\upmu \text{m}$ each mounted on a custom-designed 3d printed plastic frame. An oscillating voltage on the blades was provided with the function generator (Tektronix AFG1022) and amplified with power operational amplifier (Apex PA95).

### Interferometric probes

Both iLenses were biconvex lenses of focal lengths $f=5~\text{cm}$ each, coated with 60:40 intensity reflectivity on one side. The probe laser was a linearly polarized, 10 mW He-Ne laser of 632 nm wavelength (Thorlab HNL100L). The laser power was controlled on liquid surface by a 10 cm diameter circular ND filter wheel (Thorlabs, OD-4) and measured with a power meter (Thorlabs, Model PM100D). Laser spot size on liquid surface was measured with beam profiler (Thorlabs, BP106-VIS).

### Data acquisition

A fast photodiode (1 ns rise time, Thorlabs, DET10A) and a 2.5 GS/s oscilloscope (DPO 3054) measured the central fringe intensity. For a precise measurements of probe positions, linear motorized translation stage (Thorlab MTS50-Z8) having travel range 50 mm and minimum increment $0.05~\upmu \text{m}$ was used. The whole experimental setup was assembled on a Thorlab optical table floating in air and covered with an enclosure to minimize stray noise. The ambient temperature was measured to be $20\pm 1 \, ^{\circ } \text{C}$ with relative humidity $\sim 55\pm 5\%$.

## Supplementary Information

Supplementary Information.
