# Supplementary material for: Universal Stokes’s nanomechanical viscometer
Source: Sci Rep. 2021 Jul 13;11:14365. doi: 10.1038/s41598-021-93729-0 (PMC8277784; doi:10.1038/s41598-021-93729-0)
Supplement: Supplementary file 1 — Supplementary Information. [file 41598_2021_93729_MOESM1_ESM.pdf]

Supplementary: Universal Stokes's nanomechanical viscometer

Komal Chaudhary, Pooja Munjal and Kamal P. Singh
Department of Physical Sciences, Indian Institute of Science Education and Research Mohali, Sector-81, Manauli 140306, India.

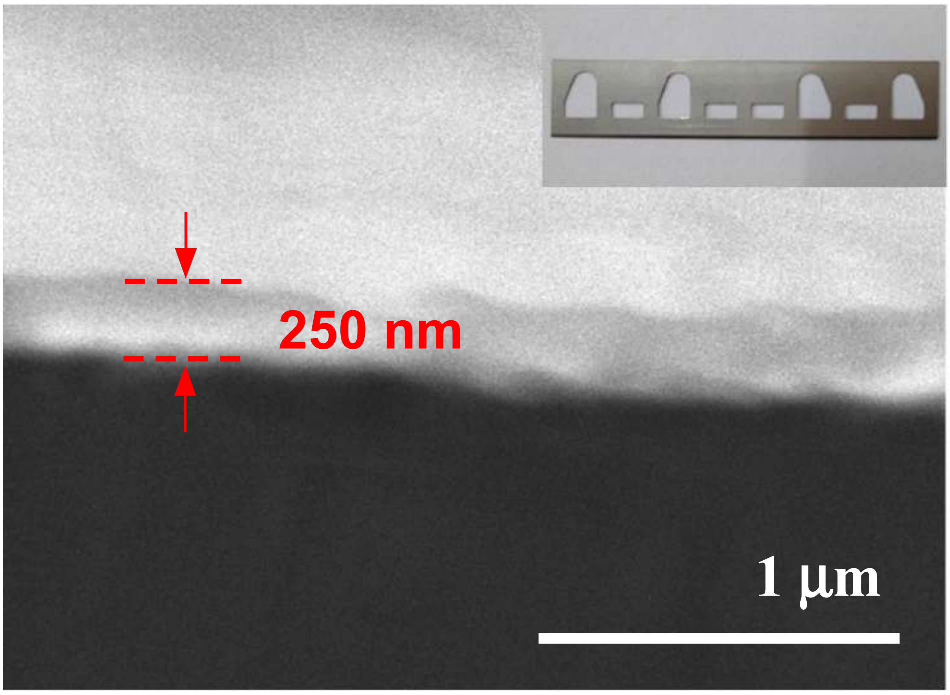

Fig. S1: Electron microscope image showing edge of single metal electrode used for electrical actuation of fluids. Sharpness of the edges δw is of the order of $\sim 250\ nm$. Inset: optical image of the blade. Five such blades were put together to make a comb-like structure to produce enhanced electric field.

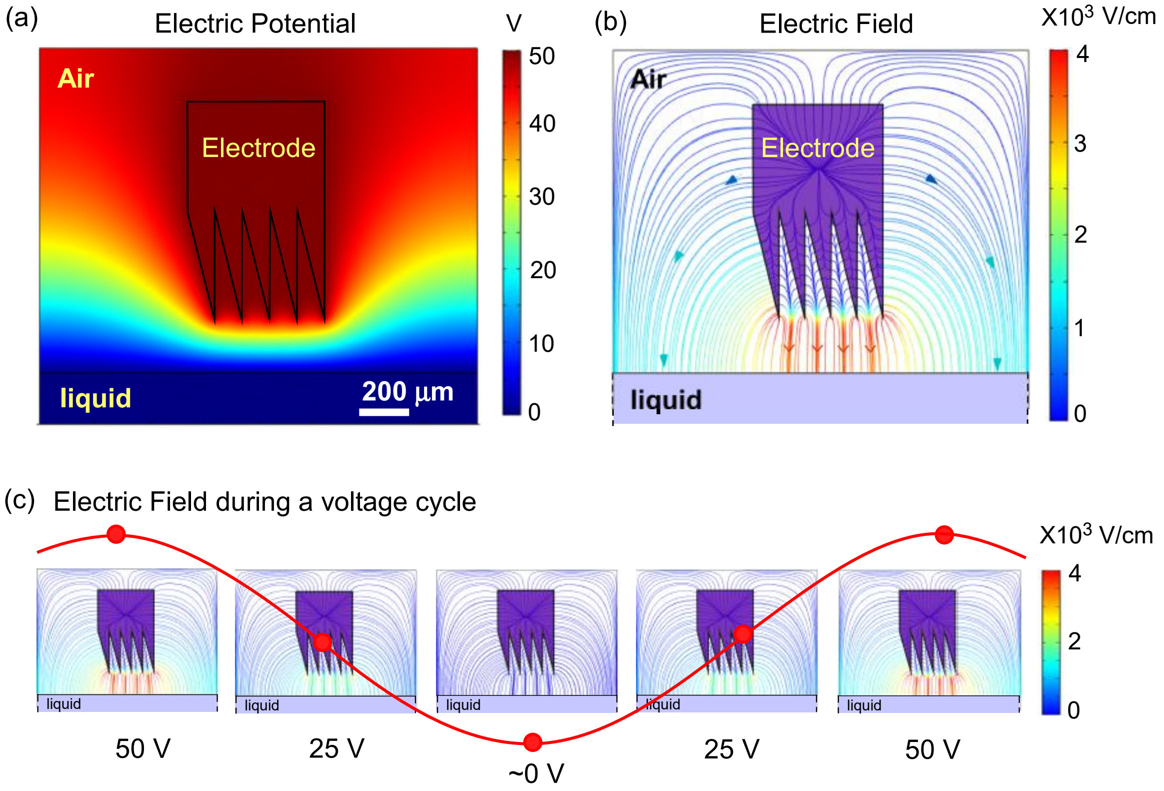

Fig. S2: Comsol simulation of electric potential and field lines in the vicinity of the excitation electrode. (a) Distribution of electric potential for an applied voltage of 50 V. (b) Electric field lines with grounded flat surface of the fluid. Electric field is locally enhanced between the electrode and fluid and is normal to the fluid surface. (c) Time-dependent electric field corresponding to a sinusoidal voltage cycle (red curve) at chosen instants (marked by filled cicles). The COMSOL model was created in 2D geometry using electrostatic module by solving $\nabla.D = \rho$ and $E = -\nabla V$. The spacing between the sharp edges making the comb was 100 μm with $\delta w = 250$ nm.

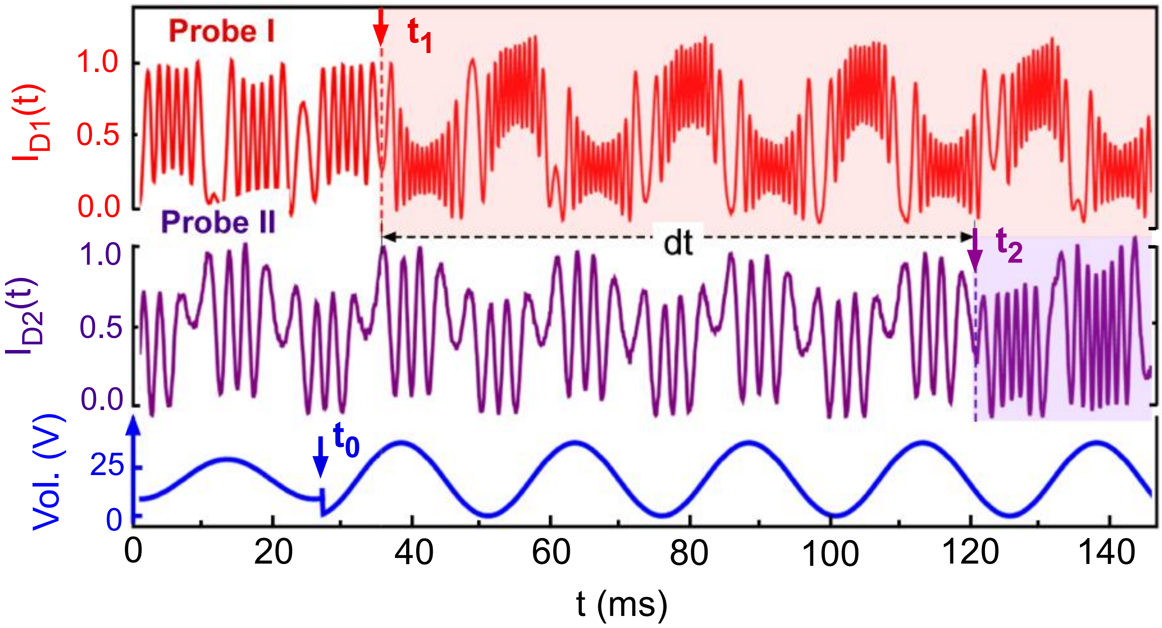

Fig. S3: Signals showing propagation of capillary waves on milk. (a) Intensities I_{D1} and I_{D2} of central fringe for Probe-I and Probe-II, respectively. The two probes are separated by $dx = 2cm$. The corresponding 40 Hz sinusoidal excitation voltage is also shown. Note that an excitation event is created at t_0 which is detected by Probe-I at time t_1 and by Probe-II at t_2 after a time delay dt.
